# Performance Assessment of Communicable Disease Surveillance in Disasters: A Systematic Review

**DOI:** 10.1371/currents.dis.c72864d9c7ee99ff8fbe9ea707fe4465

**Published:** 2015-02-24

**Authors:** Javad Babaie, Ali Ardalan, Hasan Vatandoost, Mohammad Mehdi Goya, Ali Akbarisari

**Affiliations:** Department of Disaster Public Health, School of public Health, Tehran University of Medical Sciences, Tehran, Iran; Department of Disaster and Emergency Health, National Institute of Health Research, Tehran University of Medical Sciences, Tehran, Iran; Department of Disaster Public Health, School of Public Health, Tehran University of Medical Science, Tehran, Iran; Department of Disaster and Emergency Health, National Institute of Health Research, Tehran University of Medical Science, Tehran, Iran; Harvard Humanitarian Initiative, Harvard University, Cambridge, USA; Department of Medical Entomology and Vector Control, School of Public Health, Tehran University of Medical Sciences, Tehran, Iran; Centre for Communicable Disease Management, Ministry of Health and Medical Education, Tehran, Iran; Department of Health Management and Economics, School of Public Health, Tehran University of Medical Sciences, Tehran, Iran

## Abstract

Background: This study aimed to identify the indices and frameworks that have been used to assess the performance of communicable disease surveillance (CDS) in response to disasters and other emergencies, including infectious disease outbreaks. Method: In this systematic review, PubMed, Google Scholar, Scopus, ScienceDirect, ProQuest databases and grey literature were searched until the end of 2013. All retrieved titles were examined in accordance with inclusion criteria. Abstracts of the relevant titles were reviewed and eligible abstracts were included in a list for data abstraction. Finally, the study variables were extracted. Results: Sixteen articles and one book were found relevant to our study objectives. In these articles, 31 criteria and 35 indicators were used or suggested for the assessment/evaluation of the performance of surveillance systems in disasters. The Centers for Disease Control (CDC) updated guidelines for the evaluation of public health surveillance systems were the most widely used. Conclusion: Despite the importance of performance assessment in improving CDS in response to disasters, there is a lack of clear and accepted frameworks. There is also no agreement on the use of existing criteria and indices. The only relevant framework is the CDC guideline, which is a common framework for assessing public health surveillance systems as a whole. There is an urgent need to develop appropriate frameworks, criteria, and indices for specifically assessing the performance of CDS in response to disasters and other emergencies, including infectious diseases outbreaks. Key words: Disasters, Emergencies, Communicable Diseases, Surveillance System, Performance Assessment

## Introduction

Disasters, whether natural or man-made, are common events worldwide^1^ . These events kill and injure people, destroy health facilities, and disrupt health systems and lifelines^2^ . Disasters displace populations and interrupt routine communicable disease management (CDM) programs, including surveillance systems and immunization programs^3^. In the conditions that develop after disasters, populations are very vulnerable to outbreaks of communicable diseases, and there are many examples of communicable disease outbreaks after disasters 4. These include cholera in Haiti after the 2010 earthquake, malaria after floods in Brazil, dengue fever after floods in the Dominican Republic, and acute diarrhea after the 2005 Pakistan earthquake 5. Accordingly, CDM has become one of the most important components of health care programs in disaster response management 4. The most urgent task in CDM is the establishment of a surveillance system (SS) for timely detection of any increase in disease occurrence and the introduction of rapid control measures. Almost, all of health systems establish a SS in response to disasters 6 ^,^ 7 .

The establishment of an effective SS in a disaster or emergency setting is a complex and difficult process requiring a large number of resources including manpower, equipment, and administrative facilities. To determine whether CDS meets target goals, it is necessary to measure the performance of these SS 8.

An appropriate and unique assessment system that is established according to disaster characteristics is required for the monitoring of SS. An effective assessment system should include appropriate indices and should be conducted in a correct method. Such measures can improve health system response as well 9.They can define shortcomings and provide a guide for future-response success.

Management experts believe that “what cannot be measured cannot be managed.” Therefore, the measurement of performance is considered one of the most important components of efficacy and effectiveness 10. In addition, according to current management knowledge, lack of an appropriate performance assessment is an important sign of weakness in a program or organization 9 .

With regard to the importance of performance assessment and its role in improving the performance of communicable diseases SS in response to disasters, there are 2 main questions:

What kind of performance assessment frameworks, indices, and criteria currently exist for CDS systems in response to disasters and other emergencies, including infectious diseases outbreaks? What are the characteristics of these frameworks, indices, and criteria? The aim of this systematic literature review was to find the answers to these questions.

## Materials and Methods

Before starting this systematic review, a written review manual was developed for searching strategy, study inclusion, and exclusion criteria. The study was then conducted according to this review process.We have reported our review according to the PRISMA guideline.

***Research questions***

The review aimed to answer the following questions:

1. What kind of performance assessment frameworks currently exist for CDS systems in disasters and other emergencies, including infectious diseases outbreaks?

2. What criteria and indices are used in the performance assessment of CDS systems in response to disasters and emergencies?

3. What are the characteristics of the articles on the performance assessment of CDS systems in terms of article type, study approach (qualitative or quantitative), study setting, results, hazard type, geographical location, and country or affiliation of first/corresponding authors?

***Definitions***

- For the purpose of this study a “communicable disease” is “a disease caused by living agents as infectious agents, or their products, that can be transmitted from 1 patient to another” (Synonym: infectious disease) 11.
- In the literature, “surveillance” is “the ongoing, systematic collection, analysis, interpretation, and dissemination of data regarding communicable disease for use in developing preventive actions to reduce morbidity, mortality, and to improve health” 12 .
- A “disaster” is “a man-made or natural event that disrupts the affected community functions and results in widespread losses that are greater than community resources” 13 .
- An “emergency” is “a condition that needs urgent attention and could become a disaster if not managed effectively.” In this study, both natural and man-made disasters and emergencies were included.
- Smith defines “performance assessment” as “a systematic process that seeks to monitor, evaluate, and communicate the extent to which various aspects of a system meet its key objectives” 14 .

***Inclusion criteria***

The following criteria were used to select relevant studies:

- Articles that were published in peer-reviewed journals and had addressed the performance assessment of CDS in response to disasters and emergencies (as defined above).
- Articles in any format including editorials, case reports, reviews, and original research.

***Exclusion criteria***

- All non-English articles, unless an English abstract was available.
- Papers with abstracts that were not accessible or did not include enough information for extraction of the study variables.

***Search strategy (Data sources and literature search)***

We searched 5 electronic databases, including PubMed, Scopus, Google Scholar, ScienceDirect, and ProQuest. The databases were searched for articles published up to the end of 2013. In addition grey literature[1] was searched through the "New York academy of medicine grey literature reports"^15^ . Websites of CDC and WHO searched for relevant guidelines. We also reviewed the reverences of retrieved studies to identify additional articles.

[1] - Grey literature definition: "That which is produced on all levels of government, academics, business and industry in print and electronic formats, but which is not controlled by commercial publishers."

We chose key terms and developed a search strategy based on the National Library of Medicine "Medical Subjects Headings (MeSH)".

The following search strategy was applied in the PubMed database: “(disasters [Title/Abstract]) AND surveillance [Title/Abstract], (disasters [Title/Abstract]) AND communicable diseases [Title/Abstract], (emergencies [Title/Abstract]) AND surveillance [Title/Abstract], (emergencies [Title/Abstract]) AND communicable diseases [Title/Abstract]".

To search the other databases, the PubMed search strategy was adopted. We limited our search to titles and abstracts of articles.

***Study screening process***

First, the selected key words were entered into the database search boxes, and the search was limited to abstracts and titles. The results of the key words search were reviewed by a member of the review team (JB). If the study met the inclusion criteria, it was included in the review. If there was any doubt about meeting the inclusion criteria a decision was made based on the consensus of the review team. Articles unrelated to the aim of the present study were excluded. The remaining titles were entered into an Excel spreadsheet and sorted. Duplicates were excluded. Next, the abstracts of the related titles were screened for their precise relevance to the aims of the present study. If an abstract met the inclusion criteria, it was included in the review. Abstracts that were not precisely relevant were excluded. The remaining papers were included in the review. The full texts of these articles were downloaded from the databases. If an article was not available free of charge, we paid for access. Two papers did not have full text that was accessible to us, and the study variables were not extractable from the abstracts, so they were excluded from the review.

***Data analysis***

The finally included papers were evaluated by a member of the review team (AA) using a data abstraction sheet developed by the research team. This data sheet included the study variables: name of the journal; name of the first author; number of authors; publication year; type of potential hazard; model/framework used for the CDS performance assessment; indices/criteria and tools used for the CDS performance assessment; the study approach; and the location of study. In the extraction of CDS performance assessment criteria, indices, and the study approach, our first priority was the authors’ statement. If criteria, indices, and the study approach were mentioned in the article, the data were included in our data abstraction sheet. If not, the review team used a consensus approach to decide whether the data should be included.

***Ethics and dissemination*** **

Ethical approval was not required for this literature review.

## Results

***Literature search***

The initial search strategy resulted in a total of 3928 articles/documents (3902 resulted from database searching and 26 documents resulted from Grey literature and websites searching). Of these, 3698 titles did not fulfill the inclusion criteria and were excluded, leaving 230 articles/documents that were considered potentially relevant. These papers/documents were entered into an Excel spreadsheet and sorted alphabetically. Duplicates (93 titles) were discarded. In the second phase, the abstracts of the remaining articles/documents (137 titles) were examined. In this step, 114 irrelevant abstracts were excluded and 23 papers/documents were considered for analysis. Two articles full texts were not accessible and their abstracts were not informative enough thus excluded. Four potentially relevant documents were not accessible too. In total, 16 papers and one book were included in the final review list for data extraction. Figure 1 outlines the literature search and the study selection process.

**Description of included papers:**
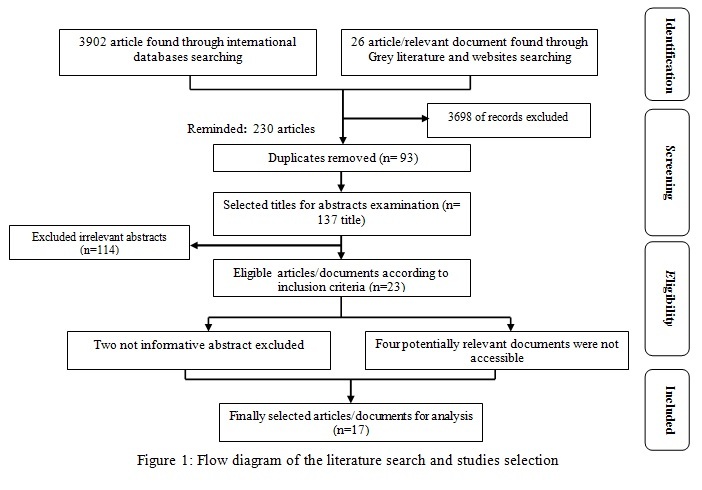

Sixteen papers and one book were included in the final review list. A total of 97 authors contributed to these 17 papers/documents. The mean number of authors per article was 5.7 (SD = 3.07). The affiliations of the first/corresponding authors of articles were the USA (n = 6, 37.5%), France (n = 2, 12.5%), the UK (n = 2, 12.5%), Australia (n = 2, 12.5%), Brazil (n = 1, 6.2%), the Netherlands (n = 1, 6.2%), and Turkey (n = 1, 6.2%). The 16 papers were published in 13 different peer reviewed journals.

The earliest article was published in 2007 and one article was published in each of 2007 and 2008. There was an increase in the number of published studies of CDS system performance assessment from 2009. For example, in both 2010 and 2011, 3 articles were published, and in 2012, 5 articles were published. The 16 studies focused on 6 hazards types including epidemics (n = 4, 25%), hurricane/cyclone (n = 2, 12.5%), heat waves (n = 2, 12.5%), mass gatherings (n = 2, 12.5), complex emergencies (n = 1, 6.2%), flood (n = 1, 6.25%), 4 article (25%) and the book included all hazards.

The studies used a quantitative approach (n = 10, 62.5%), a qualitative approach (n = 1, 6.25%), or a mixed approach (n = 3, 18.7%). Two (12.5%) studies were reviews. Six studies (37.5%) were conducted in the USA, 2 (12.5%) in France, and 2 (12.5%) in Australia. A single study was conducted in 5 countries (Brazil, Chad, Poland, the UK, and Turkey). The location of 1 (6.2%) study was not determined.

***Results of included studies***

The 16 articles that were finally selected for review were divided into 5 groups according to the theme of the study. These 5 themes were: performance assessment of syndromic surveillance systems (31.2%); mortality/morbidity SS (25.0%); public health/disease surveillance (12.5%); the applications of cost analysis, efficacy, effectiveness, and usefulness in performance assessment of SS (25.0%); and the review of performance assessment indicators (6.2%).

The relevant book is about the communicable diseases control in emergencies, and it has a specific section for CDS in disasters.

In the reviewed articles and book, there was no specific performance assessment framework for SS in disasters. However, the CDC updated guidelines for public health surveillance system evaluation 12 was used exclusively in 3 studies. In the performance assessment of mortality, morbidity, and CDS systems 16 ^,^ 17 ^,^ 18 ^,^ 19 , the CDC guidelines were also used as part of the assessment 12 . The CDC guidelines are based on 9 criteria including simplicity, flexibility, data quality, sensitivity, positive predictive value (PPV), timeliness, acceptability, representativeness, and stability.

Of the CDC public health surveillance evaluation attributes, the most widely applied was timeliness, which was used in 7 studies. Flexibility was used in 5 studies, data quality in 4, simplicity in 3, stability in 3, and usefulness and representativeness in 2 studies. In all cases, timeliness, data quality, sensitivity, specificity, PPV, cost, and representativeness were calculated quantitatively. Flexibility, usefulness, simplicity, and acceptability were calculated in a qualitative manner. Stability was calculated both quantitatively and qualitatively.

In the reviewed book^20^ 10 indicators suggested for PA of SS including: zero reporting, completeness, timeliness, the number of cholera cases for which samples were confirmed by the laboratory, the number of malaria cases confirmed by blood smear date of onset of the first case, date of reporting using outbreak alert form, date of investigation, date of response. These findings are presented in Table 1.

Overall, in the 16 articles and one book that were included, 31 criteria/measures and 35 indicators were used or suggested for the assessment/evaluation of the performance of CDS systems in response to disasters and emergencies.

**Table 1: Summary of the reviewed articles in terms of framework/method, indicator/criteria and studied hazard for performance assessment of communicable diseases surveillance system:** 

**Study**	**Framework/method**	**Indicator/Criteria**	**Hazard**
Josseran L, Fouillet A, Caillère N, Brun-Ney D, Ilef D, Brucker G, Medeiros H, Astagneau Pv 23	CDC's updated guidelines for surveillance system evaluation	Data quality, cost, flexibility, stability, timeliness, effectiveness, sensitivity, specificity, positive predictive value	Heat wave
Zielinski A 27	Review study	Cost minimization, cost-effectiveness analysis, cost utility, cost benefit	Heat wave
Cinti S, Haas K, Paliani P, Newton D, Wright C, Zalewski C, et al. 28	Comparing with data from regional and national surveillance reports	Percentage of visits by established SS and national surveillance, percentage of samples with positive results	Pandemics
Elliot AJ, Hughes HE, Hughes TC, Locker TE, Shannon T, Heyworth J, et al. 19	CDC's updated guidelines for surveillance system evaluation (Incomplete)	Sensitivity, specificity, timeliness, data quality	Mass gathering
Hope KG, Merritt TD, Durrheim DN, Massey PD, Kohlhagen JK, Todd K, D'Este, et al. 16	CDC's updated guidelines for surveillance system evaluation(Incomplete)	Usefulness, flexibility, acceptability	Mass gathering
Schnall AH, Wolkin AF, Noe R, Hausman LB, Wiersma P, Soetebier K, Cookson ST 29	Comparing of diagnosis in developed form with ED discharge diagnosis	Agreement between discharge diagnoses and developed form	Natural hazards
Choudhary E, Zane DF, Beasley C, Jones R, Rey A, Noe RS, Martin C, Wolkin AF, Bayleyegn TM 17	CDC's updated guidelines for surveillance system evaluation	Usefulness, simplicity, flexibility, data quality, acceptability, representativeness, timeliness, stability, sensitivity, positive predictive value	Hurricane
Teixeira MG, Costa MCN, Souza LPF, Nascimento EMR, Barreto ML, Barbosa N, et al 30	Comparing Brazil's public health SS with international health regulation	Structure (legal framework and financial, human and physical resource), surveillance procedure (capacity to detect, assess, notify), response (investigate, intervene, and communicate)	Public health emergencies (reemergence of infectious disease)
Noha H. Farag, Rey A, Noe R, Bayleyegn T, Wood AD, Zane D^18^	CDC's updated guidelines for surveillance system evaluation	Simplicity, flexibility, acceptability, timeliness, stability, data quality, sensitivity, positive predictive value, representativeness	Hurricane
Bowden S. Braker K, Checchi F, Wong S. 31		Simplicity, flexibility, appropriateness, timeliness, dissemination of data	Complex emergencies
Potter MA, Sweeney P, Luliano AD, Allswede MP 32	Practice, process, outcomes (review ofpublished records)	Process (first clinical observation , accurate diagnosis, laboratory confirmation, identification of exposure source, report to public health authority, report to law enforcement authority, initiation of emergency operation plan , initation of risk-mitigation activities, initiation of past exposure prophylaxis, initiation of public health education activities, initiation of risk advice to health care workers, last reported new case); outcome indicators (primary cases, total cases, secondary cases, HCW[1]s infected) [1] - Health Care Workers (HCW)	Outbreaks
Josseran L, Caillere N, Brun-Ney D, Pottner J, Filleul L, Brucker G, et al. 33	Comparing frequency of visits due to heat related patients in 'on alert' periods (ONAP) with 'off alert' periods (OFAP)	Percentage of emergency department visits, percentage of hospitalization, incidence of heat related diseases	Heat wave
Stoto MA 34	‘‘Triangulation’’ approach	Validity, utility	Pandemics
Hope K, Merritt T, Eastwood K, Main K, Durrheim DN, Muscatello D, Todd K, Zheng W 35	Comparing traditional SS with Syndromic SS	Timeliness	Natural disasters
Rosenkötter N, Ziemann A, Riesgo LGC, Gillet JB, Vergeiner G, Krafft T, Brand H 24	CDC's updated guidelines for surveillance system evaluation(Incomplete)	Validity, timeliness, sensitivity, specificity	Pandemics
Akgun D 36	Case report	Water sanitation, immunization, organization of health services, public education	Flood
Conolly MA (Editor). 20		Zero reporting, completeness, timeliness, the number of cholera cases for which samples were confirmed by the laboratory, the number of malaria cases confirmed by blood smear date of onset of the first case, date of reporting using outbreak alert form, date of investigation, date of response	All hazards

## Discussion

The aims of this research were to review and extract the frameworks and indices that have been used for assessing the performance of CDS systems in response to disasters and emergencies. An extensive systematic literature review was conducted using 5 popular international databases. Our initial search resulted in 3902 titles. Finally, 17 studies/documents met the study inclusion criteria and were considered in the data abstraction list.

The first disaster-related article was published in 1945, but the first article related to the performance assessment of CDS in disasters was not published until 2007. Surprisingly, there was a 62-year gap between the 2 publications.

Searching for the term “disaster” in PubMed resulted in more than 60,261 papers, but we could only find 16 papers about CDS systems performance assessment in response to disasters and emergencies. Therefore, performance assessment in this field is a new research focus, although in recent years there has been a growing interest in CDS system performance assessment in response to disasters. With a recent increase in the number of disasters, many countries have spent millions of dollars improving the response of their health care systems. Consequently, the assessment of the efficacy and effectiveness of the response to disasters has become a focus of investigation, to ensure that resources are used in the most efficient and effective way 9 ^,^ 10 ^,^ 11 ^,^ 12 ^,^ 13 ^,^ 14 ^,^ 15 ^,^ 16 .

According to current management knowledge, lack of an appropriate performance assessment system is a symptom of weakness and disorder in an organization or program 9 . For this reason, there has been growing interest in the performance assessment of organizations and programs in recent years 21 . Although there are several performance assessment frameworks documented in the literature, in our review we were unable to find any established framework for assessment of this specific field (SS in disasters). However, the US-based CDC has developed guidelines for the evaluation of public health surveillance systems, but only 3 studies used those guidelines exclusively. A number of other studies used this framework in part 22 . This suggests that there is no consensus on the application of the CDC guidelines for the performance assessment of CDS in disasters.

Furthermore, some economic assessment criteria have been suggested for use in the performance assessment of SS in disasters, but these criteria have not been used in practice.

The selection of appropriate criteria and indexes for CDS assessment is essential to meaningful research. In the included studies, 31 criteria/indices were used and the attributes suggested by CDC were the most widely used criteria. Some studies used other criteria and indices in addition to those of CDC 17 ^,^ 22 ^,^ 23 . This is another indication that there is no clear agreement on the application of the CDC guidelines for the performance assessment of CDS in disasters.

Different definitions have been proposed for the CDC criteria, and different scientific methods (qualitative and quantitative) have been applied for measurements. For example, Josseran divided the timeliness criterion into 4 steps and defined it as “the time that it takes for data to be collected, processed, analyzed, and publicized” 23 . Choudhary defined timeliness as “the average time that a death is reported by a surveillance system” 17 , while Farag defined the same term as “the speed of data transmission between surveillance system steps” 18 . In the article by Josseran, sensitivity, specificity, and PPV were suggested as components of the effectiveness of surveillance systems 23 , while in another study the same indices were used for validity 24 . In the study by Farag, validity was based on data quality 18 . These discrepancies show that there is a lack of consistency in the assessment of this specific field.

Despite the reasonably wide spread use of the CDC guidelines for systems performance assessment in response to disasters and emergencies, experts have emphasized that there are no generally accepted metrics 3 ^,^ 9 ^,^ 25 . Many scientists, universities, and research centers have identified this deficiency, and it has been a research priority for many years 1 ^,^ 2 ^,^ 26 . However, there is still an urgent need for the development of universal frameworks, methodologies, metrics, and criteria for the performance assessment of CDS systems in response to disasters and emergencies.

**Limitations**

This review has some limitations. During the study period, the Web of Science (ISI) was not available, and was excluded from the search process. Studies were only included if the texts or abstracts were available in English. Therefore, there is a bias in the selection of studies. However, high sensitivity was used in the database search, yielding more than 3928 titles.

Another limitation was the identification of criteria and indices. We addressed this problem by using a consensus approach between reviewers. However, there were some errors in the determination of criteria and indicators.

Finally, there was limited access to the full text of some papers and four potentially relevant documents were not accessible. We were unable to extract the study variables from 2 articles and those results were excluded from the study.

**Conclusion**

Performance assessment is an integral component of the management of all organizations and the lack of performance assessment is considered a significant sign of weakness in an organization. Therefore, the lack of accepted mechanisms for the assessment of the performance of CDS systems in response to disasters is an important weakness. Although some studies attempted to assess surveillance systems, the results of this systematic literature review suggest that there is no clear and comprehensive assessment framework in place. While the CDC framework has been used in some studies, it is not specific for SS in response to disasters. Some criteria and indices were widely applied, but were not universally accepted, and researchers used different definitions and scientific approaches. Further research is required to identify and develop a universally accepted framework for the assessment of CDS systems in response to disasters and emergencies.

## Correspondence

Ali Ardalan, MD, PhD. Department of Disaster Public Health, School of Public Health, Tehran University of Medical Science, Tehran, Iran.

Email: aardalan@tums.ac.ir

## Appendix 1

### PRISMA Checklist

Download PDF
